# Kinome and phosphoproteome reprogramming underlies the aberrant immune responses in critically ill COVID-19 patients

**DOI:** 10.1186/s12014-024-09457-w

**Published:** 2024-02-22

**Authors:** Tomonori Kaneko, Sally Ezra, Rober Abdo, Courtney Voss, Shanshan Zhong, Xuguang Liu, Owen Hovey, Marat Slessarev, Logan Robert Van Nynatten, Mingliang Ye, Douglas D. Fraser, Shawn Shun-Cheng Li

**Affiliations:** 1 Departments of Biochemistry, Western University, London, ON N6A 5C1 Canada; 2 Department of Pathology and Laboratory Medicine, Western University, London, Canada; 3 Departments of Medicine and Pediatrics, Western University, London, Canada; 4 CAS Key Laboratory of Separation Sciences for Analytical Chemistry, National Chromatographic R&A Center, Dalian Institute of Chemical Physics, Chinese Academy of Sciences (CAS), Dalian, 116023 China; 5 Lawson Health Research Institute, 750 Base Line Rd E, London, ON N6C 2R5 Canada

**Keywords:** COVID-19, SARS-CoV-2, Mass spectrometry, Tandem mass tag, Proteome, Phosphoproteome, Kinome reprogramming, Immune regulation, Cytokine, Antibody response

## Abstract

SARS-CoV-2 infection triggers extensive host immune reactions, leading to severe diseases in certain individuals. However, the molecular basis underlying the excessive yet non-productive immune responses in severe COVID-19 remains incompletely understood. In this study, we conducted a comprehensive analysis of the peripheral blood mononuclear cell (PBMC) proteome and phosphoproteome in sepsis patients positive or negative for SARS-CoV-2 infection, as well as healthy subjects, using quantitative mass spectrometry. Our findings demonstrate dynamic changes in the COVID-19 PBMC proteome and phosphoproteome during disease progression, with distinctive protein or phosphoprotein signatures capable of distinguishing longitudinal disease states. Furthermore, SARS-CoV-2 infection induces a global reprogramming of the kinome and phosphoproteome, resulting in defective adaptive immune response mediated by the B and T lymphocytes, compromised innate immune responses involving the SIGLEC and SLAM family of immunoreceptors, and excessive cytokine-JAK-STAT signaling. In addition to uncovering host proteome and phosphoproteome aberrations caused by SARS-CoV-2, our work recapitulates several reported therapeutic targets for COVID-19 and identified numerous new candidates, including the kinases PKG1, CK2, ROCK1/2, GRK2, SYK, JAK2/3, TYK2, DNA-PK, PKCδ, and the cytokine IL-12.

**Supplementary Information:**

The online version contains supplementary material available at 10.1186/s12014-024-09457-w.

## Introduction

The severe acute respiratory syndrome coronavirus-2 or SARS-CoV-2 has infected more than half a billion people and claimed more than 6 million lives worldwide to date. While the majority of individuals infected by the coronavirus, including emerging variants of concern (VOC), have mild symptoms or are asymptomatic, 5–10% develop severe diseases that require hospitalization [1]. Patients with severe symptoms usually develop acute respiratory distress syndrome (ARDS) [2] and/or sepsis, which are major causes of morbidity and mortality. The coronavirus disease 2019 (COVID-19) pandemic has sparked an unprecedented effort from the scientific community to understand the disease mechanism and developing therapeutic and immunization strategies, culminating in the approval of several vaccines and antiviral drugs for emergency use by the regulatory bodies in the US and Europe [3, 4].

Despite these phenomenal achievements, the molecular underpinnings of severe COVID-19 have not been fully elucidated. Studies to date have shown that the SARS-CoV-2 infection elicits a wide range of aberrant biochemical and cellular changes that are rooted in a defective and often excessive immune response to the virus [5]. For example, while cytokines play an important role in antiviral immunity, rapid production of a large quantity of proinflammatory cytokines, referred to as the cytokine release syndrome (CRS), is associated with severe COVID-19 cases [6]. Furthermore, severe diseases are frequently characterized with lymphopenia or reduced numbers of circulating T cells, B cells or/and natural killer (NK) cells [7]. Nevertheless, immune profiling has revealed activation of a subset of T cells or extrafollicular B cells and production of neutralizing antibodies in severe COVID-19, suggesting that these patients are capable of mounting cellular and humoral immune responses [8]. Intriguingly, some patients with a strong antibody response early do poorly in controlling the infection and ultimately succumb to the disease [9]. This dichotomy highlights deficiencies in our understanding of the fundamental immunological processes perturbed by SARS-CoV-2 [10].

To identify the molecular, cellular, and immunological abnormalities of COVID-19 in a systematic and unbiased manner, we employed quantitative mass spectrometry (MS) and complementary biochemical assays to characterize the peripheral blood—the barometer of the immune system. By comparing the peripheral blood mononuclear cell (PBMC) proteome and phosphoproteome of sepsis patients in the intensive care unit (ICU) who tested positive or negative for the SARS-CoV-2 virus with age- and sex-matched healthy subjects, we identified the protein and phosphoprotein signatures and the regulatory/signaling pathways that characterize severe COVID-19. We show that the sepsis patients with or without SARS-CoV-2 infection share many common characteristics, including dysregulated immune signaling. Intriguingly, the two groups of ICU patients exhibited distinct cytokine profiles. Our work has identified numerous potential therapeutic targets for the development of targeted immunomodulatory therapies for the treatment of patients with severe COVID-19 diseases.

## Methods

### Study design and blood sample collection

Patients were admitted to the level-3 academic intensive care unit (ICU) at the London Health Sciences Centre-Victoria Campus (London, Ontario) and were suspected of having COVID-19 based on standard hospital screening procedures. Blood samples were collected starting at admission for COVID-19^−^ patients, or on days 1, 7 and 10 for COVID-19^+^ patients in April–May 2020. COVID-19 status was confirmed by detection of two SARS-CoV-2 viral genes using polymerase chain reaction. Although ICU severity of illness scores have not been validated in COVID-19 ^+^ patients, we calculated multiple organ dysfunction score (MODS) and Sequential Organ Failure Assessment (SOFA) score for the patients. Final participant groups were constructed by age- and sex-matching COVID-19 positive and negative ICU patients (Additional file 2: Tables S1-3), as well as healthy controls that had blood samples previously banked in the Translational Research Centre (directed by D. Fraser, https://translationalresearchcentre.com/).

The peripheral blood mononuclear cell (PBMC)/buffy coat and plasma samples were de-identified prior to transfer from the hospital to a biosafety Level 3 (CL3) lab (ImPaKT, Western University) following Transportation of Dangerous Goods (TDG) guidelines. All plasma samples were heat-inactivated at 56 ℃ for 30 min and the PBMCs were lysed in 9 M Urea in HEPES buffer (20 mM HEPES, 1 mM sodium orthovanadate, 10 mM NaF, pH8.0) at the ImPaKT CL3 facility as per Western University biosafety regulations. Heat-inactivated plasma and lysed PBMCs samples were verified free of the virus before they were transferred to the testing laboratory.

### Pervanadate treatment of PBMCs for the pTyr booster channel

We used the pervanadate boost method [11] to increase identification of tyrosine phosphorylated peptides with the isobaric TMT labelling experiments. The pervanadate solution was prepared by adding 10 μl of 0.1 M sodium orthovanadate to 10 μl of 0.2 M hydrogen peroxide (diluted 50 × from a 30% stock). The solution was then incubated at room temperature for 15 min and was added to the PBMCs in PBS. A part of PBMCs from healthy donors were treated with 0.1 mM pervanadate solution in phosphate-buffered saline (pH 7.4) at 37 ℃ for 10 min.

### Sample processing for proteomics and phosphoproteomics analyses by mass spectrometry

#### Hemoglobin depletion and protein precipitation

Hemoglobin was depleted from PBMC whole cell lysate samples according to HemogloBind (Biotech Support Group LLC) manufacturer instruction with modifications. Briefly, 10 ml HemogloBind beads were added to 1 ml whole cell lysate, and the mixture was vortexed for 10 min at room temperature. The mixture was then centrifuged for 5 min at 10,000 rpm and the protein supernatant was collected and precipitated with 5 volumes of ice-cold acetone/ethanol/acetic acid (v/v/v/ = 50/50/0.1) at – 20 ℃ overnight. Protein pellets were collected by centrifugation at 17,000 *g* for 20 min the following day and the resulting pellets were washed with ice-cold 75% ethanol once and centrifuged at 17,000 *g* for 3 min. Ethanol was removed and pellets were dried briefly and then resuspended in urea lysis buffer (9 M urea, 20 mM HEPES, 1 mM sodium orthovanadate, 10 mM NaF, pH8.0).

#### Protein processing and digestion

Protein concentration was estimated by Bio-Rad protein assay kit. The protein concentration was adjusted to 8 µg/µl in urea lysis buffer and reduced with 10 mM dithiothreitol (DTT) for 1 h at room temperature. Protein was then alkylated with iodoacetamide (IAA) to a final concertation of 28 mM IAA followed by incubation for 45 min in the dark at room temperature. Protein solution was then diluted 1:3 (vol/vol) with digestion buffer (50 mM HEPES, 1 mM orthovanadate, 10 mM NaF, pH 8.0) to decrease urea concentration, LysC was then added in a ratio of 1 mAU per 50 µg of total protein followed by incubation for 2 h at 25 ℃ with gentle shaking. Trypsin was then added at a 1:50 ratio, and incubated overnight at 28 ℃. The resulting peptide was desalted using SepPak C18 cartridges (Waters WAT054955) and SpeedVac-dried.

#### Tandem Mass Tag (TMT) labelling

For mass spectrometry analysis, we labelled 25 patient or healthy control samples with the 11-plex TMT isobaric labelling reagent (ThermoFisher Scientific A37725) (see Additional file 2: Table S4 for sample identities with TMT set/channel numbers). In addition, we employed the pervanadate boost channel approach by including the pervanadate-treated PBMCs in channel 1 of each 11-plex sample. Three sets of 11-plex reagents were used to label all samples. The TMT labelling procedure was modified from [12]. The desalted peptides were reconstituted in 0.1% formic acid to determine peptide concentration by the BCA protein assay kit (Pierce 23,225). Portions of 200 µg peptides from each sample were aliquoted and vacuum dried. Each of 0.8 mg 11-plex TMT labelling reagents was reconstituted in 41 µl acetonitrile. The peptides were reconstituted in 40 µl of 50 mM HEPES (pH 8.5) to prepare 5 mg/ml peptide solution and were then mixed with 20.5 µl of the TMT reagent prepared above. The labeling reaction was allowed to proceed for 2 h at room temperature before a 1 µl aliquot was taken from each sample to determine the TMT labelling efficiency by mass spectrometry. The reaction was quenched by adding 4 µl of 5% hydroxylamine. The 11 samples were combined (for a total of 2.2 mg peptides) and desalted using a SepPak C18 cartridge.

For enrichment of pTyr peptides, the SH2-Superbinder (SH2S) agarose beads (Precision Proteomics, London, Canada) were used. The TMT-labelled peptides were reconstituted in 50 mM ammonium bicarbonate and incubated with the SH2S beads for 30 min at room temperature with rotation. The flow-through fraction was saved for later use. The beads were washed four times with the same buffer. The bound pTyr peptides were eluted by 0.4% trifluoroacetic acid (TFA). The eluted peptides were loaded onto a High pH Reverse Phase column (Pierce 84,868). The peptides were eluted into eight fractions which were concatenated into four vials for MS injections.

For the flow-through peptides not captured by the SH2S beads, a 100-µg portion was separated into 12 fractions by the High-pH fractionation kit for proteome analysis. A further 500 µg portion was used for phosphopeptide enrichment using the Ti^4+^-immobilized metal affinity chromatography (IMAC) resin following a published protocol [13]. Briefly, a 500-µg portion of the flow-through fraction from the SH2S enrichment step was mixed 1:1 (v/v) with 80% acetonitrile/6% TFA solution and then loaded to the IMAC resin. After incubation and wash steps (wash-1 solution: 50% acetonitrile, 6% TFA, 200 mM NaCl; wash-2 solution: 30% acetonitrile, 0.1% TFA), the peptides were eluted by 10% ammonia, and dried by Speedvac. The dried phosphopeptides were separated into eight fractions and then concatenated into four vials for MS injections.

#### LC–MS/MS experiments

The fractionated peptides were reconstituted in 2% acetonitrile/0.1% formic acid (FA). The peptides were analyzed by the data-dependent acquisition method on a Q-Exactive Plus mass spectrometer coupled to an the EASY-nLC 1000 system (ThermoFisher Scientific). The peptides were separated on an EASY-Spray ES803A C18 analytical column (75 µm diameter, 500 mm long, ThermoFisher Scientific) at a flow rate of 300 nl/min with a linear gradient from 3 to 40% acetonitrile in 0.1% formic acid. The gradient length was 2 h for pTyr phosphoproteome fractions, 4 h for proteome, and IMAC phosphoproteome fractions. See Additional file 2: Table S5 for mass spectrometry data acquisition parameters.

Peptide identification and quantification were performed using FragPipe version 17.1 [14]. The mass spectra were searched against the human SwissProt sequences (20,409 entries, downloaded on December 2, 2021) and their corresponding decoys, supplemented with common contaminants. The desired protein FDR was set to 0.01. For proteome data processing, the TMT10 workflow was loaded and the plex was changed to 11-plex. Trypsin was specified as the proteolytic enzyme with up to two missed cleavage sites allowed. For IMAC- and SH2S-enriched datasets, the TMT10-phospho workflow was used, which includes phospho(STY) as an additional variable modification. For phosphoproteomic data, the minimal peptide length for the search was set as 6, whereas the value was set as 7 for proteome data. The virtual reference was used for the TMT multi-batch normalization. The median centering normalization was used for normalization between the sample channels. Other Fragpipe parameters are left at default values. The processed proteome and phosphoproteome data can be found in Additional file 2: Tables S6-7.

#### Proteome and phosphoproteome data analysis

For data analysis, only the proteins (for proteome) or phosphosites (for phosphoproteome) observed in at least three samples in each of the five groups (COV-D1, D7, D10, ICU, HC) were retained. Phosphosites with the localization probability > 0.75 were retained. Perseus version 1.6.14.0 was used to analyze the data [15]. The VolcaNoseR server was used for drawing volcano plots [16]. The list of the human kinases was based on [17]. The ITRM motifs were based on a previously identified list [18]. The KSEA App was used to predict active kinases [19]. The Metascape server was used for functional gene annotation analysis [20]. The heatmaps were prepared with the Morpheus server.

### RNA isolation and quantitative polymerase chain reaction (qPCR)

Isolation and purification of total RNA from the PBMCs were carried out according to RNeasy^®^ Mini Kit followed by cDNA preparation using reverse transcriptase and random primers. The qPCR amplification was performed with primers specific for the cytokines/chemokines of concern. After 40 cycles of PCR, ΔCt values were determined using different cytokine and chemokine primers. Differences in mRNA levels were then calculated using the 2 ^−^ (ΔΔCt) method. The expression of β-actin was used to normalize mRNA content and to calculate Log2 fold change in gene expression. Samples were measured in five biological repeats and four technical repeats.

### Statistical analysis

Statistical analyses were carried out using the GraphPad Prism9 software. Unpaired One-way ANOVA was conducted to test the significance of the difference of unpaired samples from different patient groups, and repeated measure ANOVA was done for paired patients’ samples as described in the corresponding figure legends.

## Results

### MS analysis of the PBMCs reveals features of the COVID-19 proteome and phosphoproteome

Quantitative MS enabled by tandem mass tag (TMT) labelling was used to identify proteins and phosphoproteins in the blood of critically ill COVID-19 patients in comparison to age- and sex-matched SARS-CoV-2-negative sepsis patients and healthy control subjects. Specifically, the PBMCs were isolated, respectively, from the blood of 5 ICU patients who tested positive for the SARS-CoV-2 RNA (the COV group; median years of age = 61.0; IQR = 54.8–67.0), 5 SARS-CoV-2-negative ICU patients (the ICU group; median years of age = 58.0; IQR = 52.5–63.0), and 5 healthy individuals (the HC group; median years of age = 57.5; IQR = 52.8–62.8) (Additional file 2: Tables S1-3).

To gauge the proteome and phosphoproteome dynamics associated with disease progression, we included in the MS analysis serial blood samples from the COV group collected on days 1, 7 and 10 (or D1, D7, and D10) of ICU admission. Peptides from the 25 PBMC samples were labeled with TMT-11plex in three batches and subjected to liquid chromatography (LC)-tandem mass spectrometry (MS/MS) analysis (Additional file 1: Fig. S1, Additional file 2: Tables S4-5). The phosphoproteome identification was facilitated by SH2 Superbinder (SH2S)-enrichment of the pTyr-containing peptides [21] and IMAC (Additional file 1: Fig. S1). The MS analyses identified 3047 non-redundant proteins and 2437 Ser/Thr/Tyr phosphorylation sites, including 380 unique pTyr sites.

**Fig. 1:**
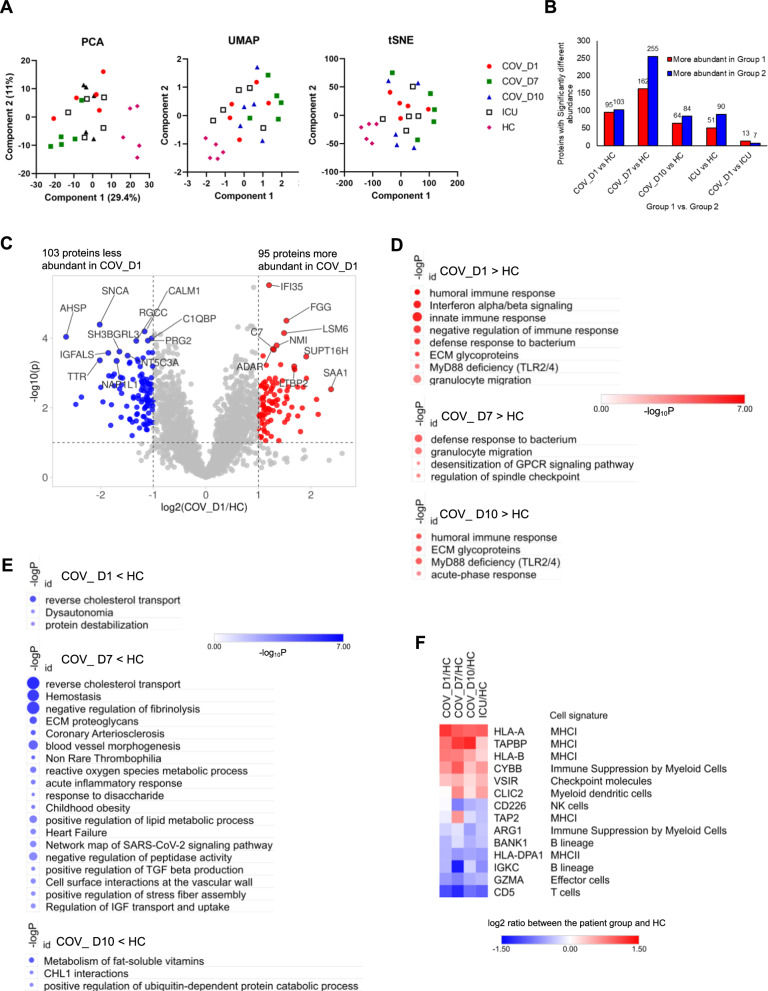
The PBMC proteome of critically ill COVID-19 patients. **A** PCA, UMAP and t-SNE plots of the proteome data showing segregation of the COVID-19 (COV) and ICU (COV-) samples away from the healthy controls (HC). **B** A bar graph showing the number of proteins with significantly increased (red) or decreased (blue) expression between the patient and HC groups. Proteins with the log2 difference > 1 and p < 0.1 between the two groups shown were considered significant. The bar graph is a summary of the differentially expressed proteins between the COVID-19^+^ day 1, 7 or 10 samples and the HC, the COV^-^ ICU samples vs. the HC, or the COV_D1 vs. ICU. **C** A volcano plot of the proteins identified in the COV_D1 samples. The significantly differentially expressed proteins (DEPs) are highlighted in red (i.e., increased expression over HC) or blue (i.e., decreased expression over HC). **D**, **E**. The enriched functions in the COV groups (**D**) or HC (**E**) identified by Metascape analysis based on the corresponding DEPs. The color of the circle denotes the p value (-logP) whereas the size of the circle is proportional to the number of proteins involved in each term. The largest number of proteins (corresponding to the largest circle) is 42. The full list of 3047 identified proteins was used as the background dataset for enrichment. Enrichment terms with -logP > 3 are shown. **F** Changes in cell-type signature proteins between the patient and the HC groups. P < 0.1 between the HC and at least one of the patient groups (student’s t-test)

Dimension reduction by Principal Component Analysis (PCA), Uniform Manifold Approximation and Projection (UMAP), and t-distributed Stochastic Neighbor Embedding (t-SNE) analyses showed clear separation of the ICU samples from the HC group (Fig. 1A). Within the COV^+^ ICU cohort, the day 7 group (COV_D7) was the most separated from the HC group whereas the day 10 group (COV_D10) appeared the most divergent. Compared to the HC, 162 proteins were significantly overexpressed and 255 significantly under-expressed in the COV_D7 group, both of which were markedly greater than between the COV_D1/COV_D10 and the HC groups (Fig. 1B). No apparent separation was observed between the COV^+^ and COV^−^ ICU samples, suggesting that the two ICU groups with suspected or confirmed sepsis (Additional file 2: Table S3) have similar proteome features. This was reinforced by the observation that only 20 proteins exhibited a significant difference in expression between the COV_D1 and ICU groups (Fig. 1B). A volcano plot of the differentially expressed proteins (DEPs) between COV_D1 and HC identified 95 proteins with significantly increased expression and 103 with significantly decreased expression in COV_D1 (Fig. 1C). Gene Ontology (GO) analysis of the DEPs identified immune responses, including humoral and innate immune responses and interferon signaling as the top upregulated biological processes in COV_D1 compared to the HC. Intriguingly, ECM glycoproteins and My88 deficiency emerged as the upregulated processes in both the COV-D1 and COV_D10 samples whereas defense response and granulocyte migration were increased in the COV_D7 group (Fig. 1D) [22, 23]. In contrast, reverse cholesterol transport, hemostasis and negative regulation of fibrinolysis were significantly downregulated in the COV_7 group (Fig. 1E) [24]. Compared to the HC, the COV (especially COV_D7) and ICU PBMCs contained more signature proteins for antigen cross-presentation through MHC-I, including HLA-A and HLA-B, TAPBP, and TAP2, but less HLA-DPA1 (MHC-II). Furthermore, we observed decreased levels for the B cell markers BANK1 and IGKC, the T cell marker CD5, the natural killer (NK) cell marker CD226, and the effector cell marker GZMA in both the COV and ICU groups compared to HC (Fig. 1F) [25, 26].

**Fig. 2:**
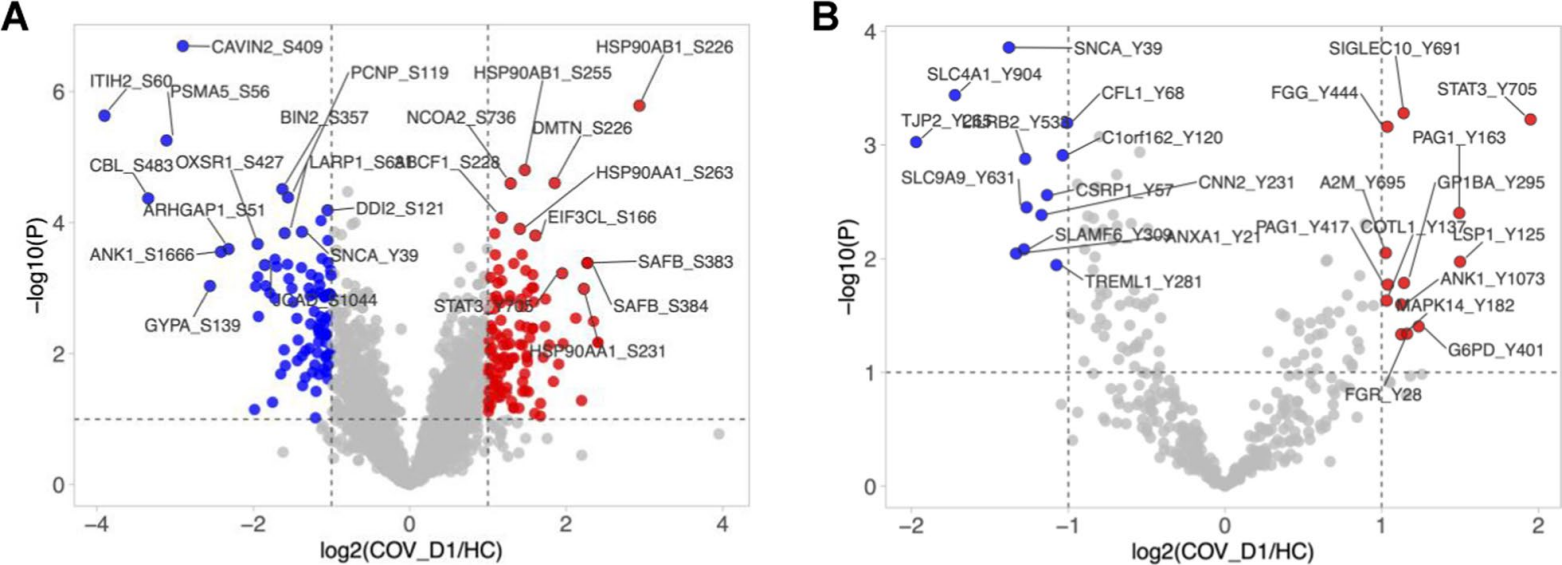
Characteristics of the COVID-19 phosphoproteome. **A** A volcano plot of 2,437 phosphosites with the ones showing a significant increase or decrease in the COV_D1 relative to the HC group (Log2(COV_D1/HC) > 1.0 or < − 1.0; p < 0.1) highlighted in red or blue. **B**. A volcano plot of the 380 identified pY sites. The significantly increased (red) or decreased (blue) sites in the COV_D1 group (compared to HC) are highlighted

Phosphorylation plays a pivotal role in intracellular signal transduction and extracellular communication with the environment. Our MS phosphoproteome analyses identified more than 2400 phosphosites, of which > 25% were significantly different between the COV and HC groups (Fig. 2A, B). A Metascape analysis of the differentially phosphorylated proteins identified fibrinolysis, integrin pathway, and response to external stimulus as significantly altered between COV (D1, D7 or D10) and HC.

### Dynamic changes of the proteome and phosphoproteome during disease progression

**Fig. 3:**
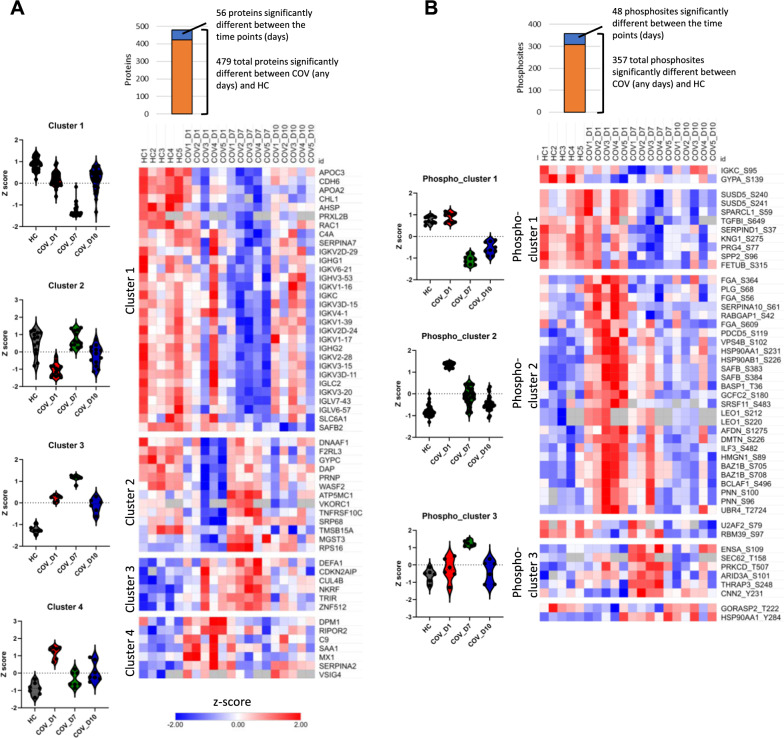
Dynamic changes in the COVID-19 proteome and phosphoproteome during disease progression. **A** Heatmap of 56 proteins with significantly different abundance between COV (anytimepoint) and HC groups, as well as between any two time points. Clusters 1 and 2 are characterized with low abundance proteins on Day7 (Cluster 1) or Day 1(Cluster 2) whereas Clusters 3 and 4 feature high abundance proteinson Day 7 (Cluster 3) or Day 1 (Cluster 4). **B** Heatmap of 48 phosphosites with significantly different abundance between COV (any time point) and HC groups, as well as between any two time points. The three main clusters are characterized by low abundanceon Day 7 and Day 10 (Phospho_cluster 1), high abundance on Day 1 (Phospho_cluster 2), or high abundance on Day 7 (Phospho_cluster 3). In **A** and **B**, the significantly regulated proteins or phosphosites were selected by two layers of filters: 1) significantly up-or downregulated between the HC and the COV groups (Day 1, 7 or 10) based on the log2 difference > 1 and p < 0.05 (unpaired T-test), and 2) significantly up-or downregulated between any 2 days, based on the log2 difference > 1 and p < 0.05 (paired T-test for each patient). See Additional file 1: Fig. S2 for detailed explanation about the filters.

Pairwise comparison identified 479 proteins and 357 phosphosites that were significantly different between the COV groups and the HC (Fig. 3, Additional file 1: Fig. S2). Intriguingly, of these differentially regulated proteins or phosphosites, 56 proteins and 48 phosphosites exhibited significant differences between the COV samples collected on different days of ICU admission. The heatmap of the 56 differentially expressed proteins (DEPs) form 4 discernible clusters (Fig. 3A). Within the Cluster 1 DEPs, the immunoglobulin genes such as IGKV, IGLV, IGHA, and IGLC constitute the majority of significantly under-expressed proteins in the COV groups, especially, in COV_D7, compared to the control, suggesting a profound defect in overall antibody production or depletion of antibodies on day 7 of ICU admission. CHD6, found down-regulated in severe COVID-19 in a recent study [27], was also significantly under-expressed in COV_D7. The Clusters 2 and 3 features DEPs over-expressed in COV_D7. Specifically, numerous proteins were significantly overexpressed on D7 than D1 or D10, including NKRF (NF-kappa-B-repressing factor) which has been reported to play a key role in SARS-CoV-2 infection [28, 29], F2RL3 (PAR4), a receptor for activated thrombin that may a role in platelets activation, and DEFA1 (neutrophil defensin 1). Finally, Cluster 4 is populated by proteins over-expressed on D1, including those involved in immune response (e.g., C9 and VSIG4, MX1) or acute phase response protein SAA1.

A heatmap of the 48 differentially phosphorylated proteins (DPPs) formed three separate clusters (Fig. 3B). Phospho_cluster 1 featured proteins over-phosphorylated on D1, including the adhesion proteins SUSD5, TGFBI, and the protease inhibitors KNG1 and SERPIND1 that are involved in coagulation. Phospho-cluster 2 featured proteins heavily phosphorylated on D1 and moderately on D7. These include regulators of pre-mRNA splicing (SRSF1, ILF3) or RNA transcription/translation (HMGN1, PNN and LEO1). Analysis of the phosphosites within Cluster-2 by S/T kinase enrichment [30] identified CK2 as one of the most active STKs (Additional file 1: Fig. S3). In contrast, one of the most highly phosphorylated/activated protein with the Phospho_cluster 3 was PRKCD (PKCδ), a kinase that plays a critical role in immune tolerance and effector functions against pathogens [31, 32]. Collectively the proteome and phosphoproteome clusters identified above not only distinguish the COV groups from the HC, but also the COV samples collected on different days of ICU admission. It is tempting to speculate that these clusters or specific DEPs/DPPs contained within them may be further developed into biomarkers for monitoring the progression of COVID-19, offering a more accurate alternative to clinical tests (Additional file 1: Fig. S4).

### Kinome reprogramming by SARS-CoV-2

**Fig. 4:**
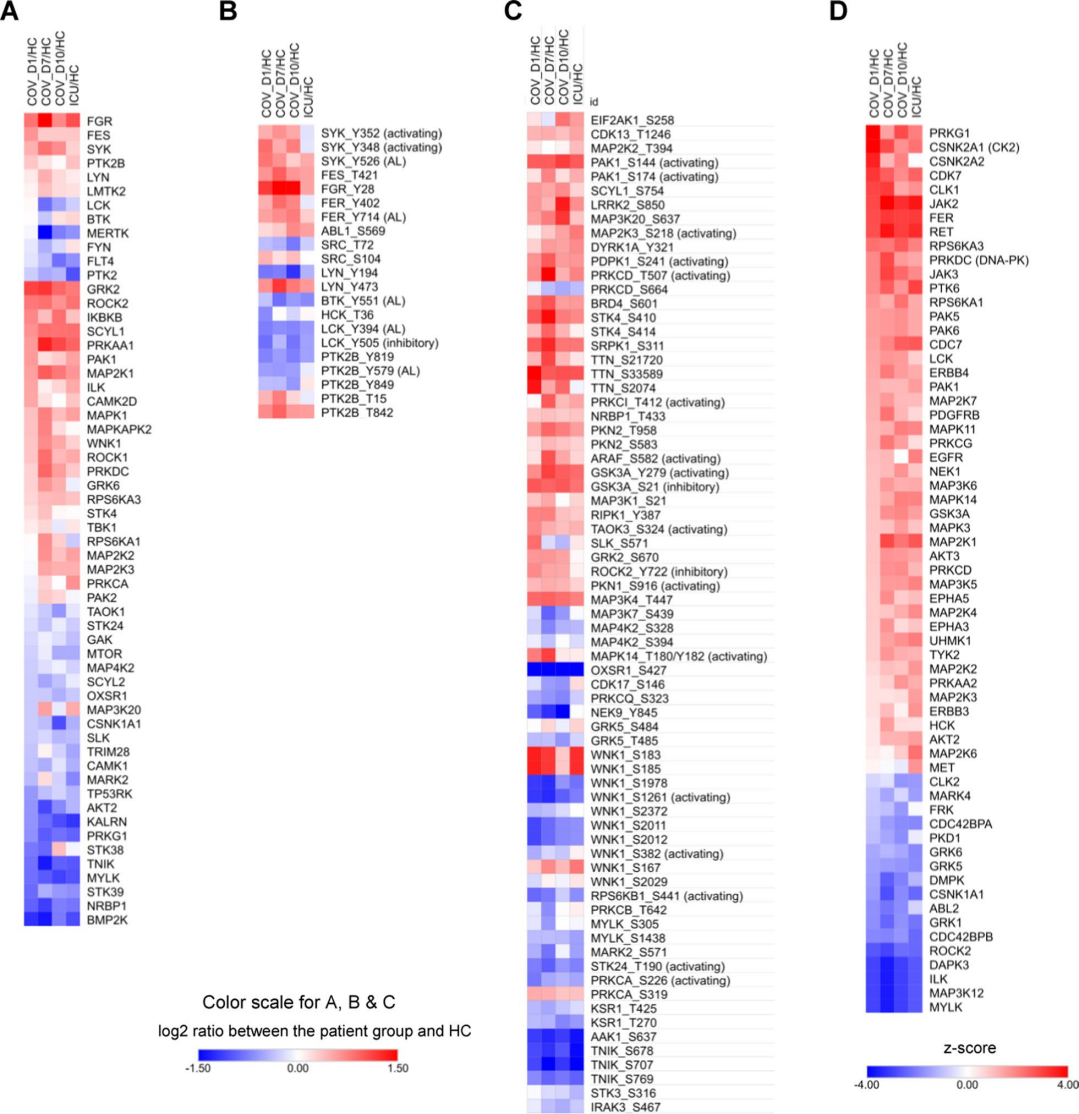
Kinome reprogramming in severe COVID-19. **A** Heatmap of significantly differentially expressed protein kinases between the patient and HC groups. **B** Differentially phosphorylated sites on Tyr kinases. **C** Differentially phosphorylated sites on Ser/Thr kinases. For **A**–**C**, p < 0.1 between HC and at least one of the patient groups, Student’s t-test. **D** Kinase activity prediction using Kinase-Substrate Enrichment Analysis (KSEA). The phosphoproteome data were used to predict which kinases may be activated or supressed in the COVID-19 PBMCs compared to healthy controls, based on enrichment of phosphorylated substrates in the COV samples. The prediction employed both the PhosphositePlus and NetworKIN datasets. Kinases with z > 1.5 or z < -1.5 are shown. “AL”: kinase activation loop, “activating”: the phosphorylation induces kinase activity, “inhibitory”: the phosphorylation inhibits kinase activity. The annotations are based on the PhosphositePlus database.

To understand the role of protein kinases in the immune responses to SARS-CoV-2, we next focused on identifying the kinases that showed a significant difference (p < 0.1) in expression between the patient samples and HC. The expression of several tyrosine kinases (TKs), including the B cell kinases LYN and SYK and the Src family kinases FGR and FES, was significantly increased in the COV and ICU groups compared to the HC. In contrast, the T cell kinase LCK and FYN were significantly decreased in expression in the COV groups. Moreover, numerous Ser/Thr kinases (STKs) were either increased (e.g., GRK2, ROCK2, ROCK1, PAK1) or decreased (e.g., BMP2K, TNIK, MYLK, and NRBP1) in expression in the patient samples, suggesting a widespread change in the kinome caused by pathogen infection (Fig. 4A).

The activity of a kinase is often regulated/induced by the phosphorylation of specific residues on the kinase, including those within the activation loop. Therefore, the phosphorylation status of the regulatory site(s) provides a facile proxy for the activity of the corresponding kinase [21]. Based on this rationale, SYK, FER, FES, and LYN were more active in COV than HC (Fig. 4B). Specifically, LYN-pY473, an activity-induced site, was increased whereas LYN-pY194, an inhibitory site, was decreased, in the COV compared to the HC group, suggesting LYN is more active in the former. Three activating pY sites, including pY526 within the activation loop, were increased in SYK, suggesting it is significantly more active in the COV samples. In contrast, the activation status of the T cell receptor (TCR) proximal kinase LCK was less clear as phosphorylation was decreased for both the activating loop Y394 and the inhibitory Y505 residues (Fig. 4B). However, the reduced LCK phosphorylation may be related to decreased LCK expression in the COV samples compared to the HC group (Fig. 4A). Intriguingly, two of the three pY sites in SYK were found decreased in the ICU group, suggesting that the B cell receptor (BCR) signaling, and B cell-mediated immune response were more robust in the COV^+^ than the COV^−^ ICU cohort (Fig. 4B). Other TKs displaying significant changes in phosphorylation included FER and PTK2B (PYK2). However, the overall kinome features were more similar than different between the COV + and COV- sepsis patients.

Approximately 30 STKs had significantly increased phosphorylation, including on activation-inducing residues, suggesting activation of these kinases (Fig. 4C). Of note, PRKCD/PKCδ, a gatekeeper of immune homeostasis [32], was significantly over-phosphorylated, especially in the COV_D7 group (Figs. 4C and 3B). The β-adrenergic receptor kinase ADRBK1/GRK2, a hallmark of cardiac stress and heart failure [33], was also highly and selectively phosphorylated in the COV PBMCs, suggesting that GRK2 may contribute to cardiac dysfunction associated with COVID-19 [34]. In support of this assertion, elevated myocardial and lymphocyte GRK2 expression and activation have been associated with heart failure [35].

Contrary to kinase activation, 16 STKs showed reduced phosphorylation in the COV samples. Of note, WNK1 exhibited significantly reduced phosphorylation on multiple sites, including the activation loop S1261 residue, suggesting that WNK1 activity is inhibited in the COVID-19 patients. As an important regulator of electrolyte homeostasis [36], WNK1 may play a role in regulating blood pressure in COVID-19 patients. The TRAF2- and NCK-interacting kinase (TNIK) is another kinase with reduced phosphorylation on multiple sites. TNIK is an essential activator of the Wnt signaling pathway, and the reduced TNIK activation, together with increased activation of GSK3A/3B [37], may collectively suppress Wnt signaling. Because Wnt signaling is involved in dendritic cell (DC) maturation and survival of regulatory T cells [38], the aberrant inactivation of TNIK and activation of GSK3A/3B may underlie the reduced Treg cell population found in hospitalized patients [39]. Moreover, TNIK is required for canonical NF-κB signaling, thus TNIK inactivation may contribute to reduced antiviral response [40].

While the MS analysis yielded quantitative phosphorylation data for numerous kinases, not all expressed kinases were detected with phosphorylation due to the stochastic nature of mass spectrometry and the unfavorable chromatographic behavior for certain phosphopeptides. Nevertheless, the activity of these kinases may be inferred by Kinase-Substrate Enrichment Analysis (KSEA). Indeed, KSEA predicted 45 kinases with increased activity and 17 with decreased activity in the COV (compared to HC) group (Fig. 4D). Of note, PRKG1 (cGMP-dependent protein kinase 1), a key regulator of nitric oxide (NO)/cGMP signaling, and CK2 (casein kinase 2), a promiscuous STK, were highly active in COV. CK2 was identified in a recent study as the top kinase activated in the Vero E6 cells following SARS-CoV-2 infection [41]. Our data underscores a critical role for CK2 in COVID-19 pathogenesis. Moreover, the Janus kinases JAK2, JAK3, and TYK2 were among the highly active kinases predicted by KSEA. Their over-activation may contribute to the autoimmune condition associated with severe COVID-19 [42]. It is also worth noting that PRKDC/DNA-PK was strongly activated in COV. Because DNA-PK deficiency may potentiate cGAS-mediated innate immunity [43], increased DNA-PK activity suggests inhibition of antiviral immune response.

### Rewiring of immune signaling in severe COVID-19

**Fig. 5:**
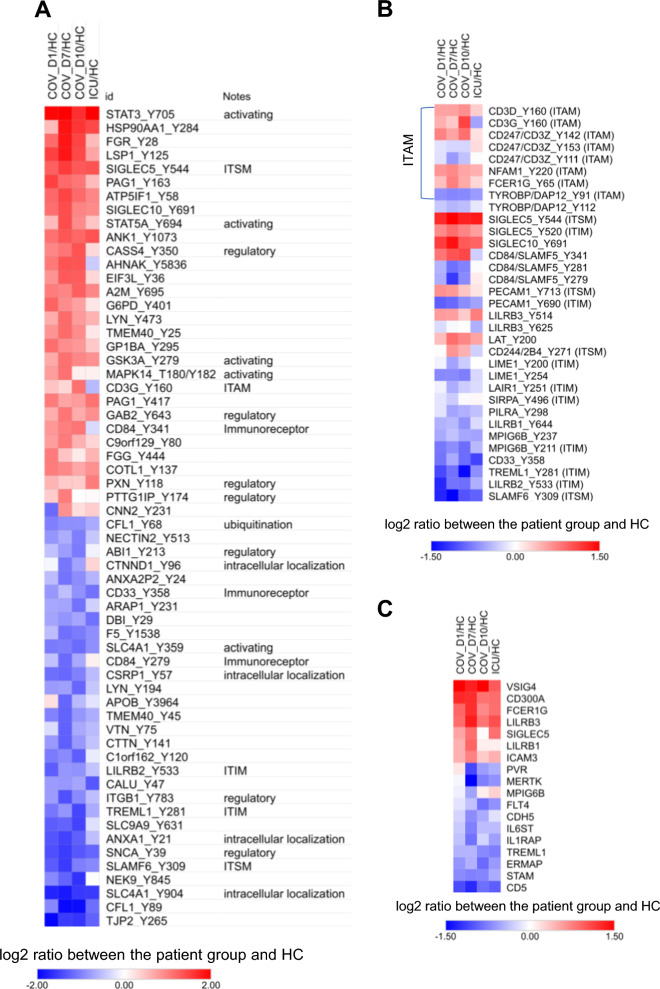
Reprograming of the immune Tyr phosphoproteome in COVID-19. **A** Heatmap of the pTyr sites that were most significantly different between the patient and HC groups. The pTyr sites with p < 0.1 and log2 difference > 1.5 fold between at least one of the patient groups and the HC are listed. **B** Heatmap of the immune receptors with significant changes in Tyr phosphorylation. **C** Differential expression of immunoreceptor proteins. p < 0.1 between the control group and at least one of the patient groups, Student’s t-test (for B and C).

**Fig. 6:**
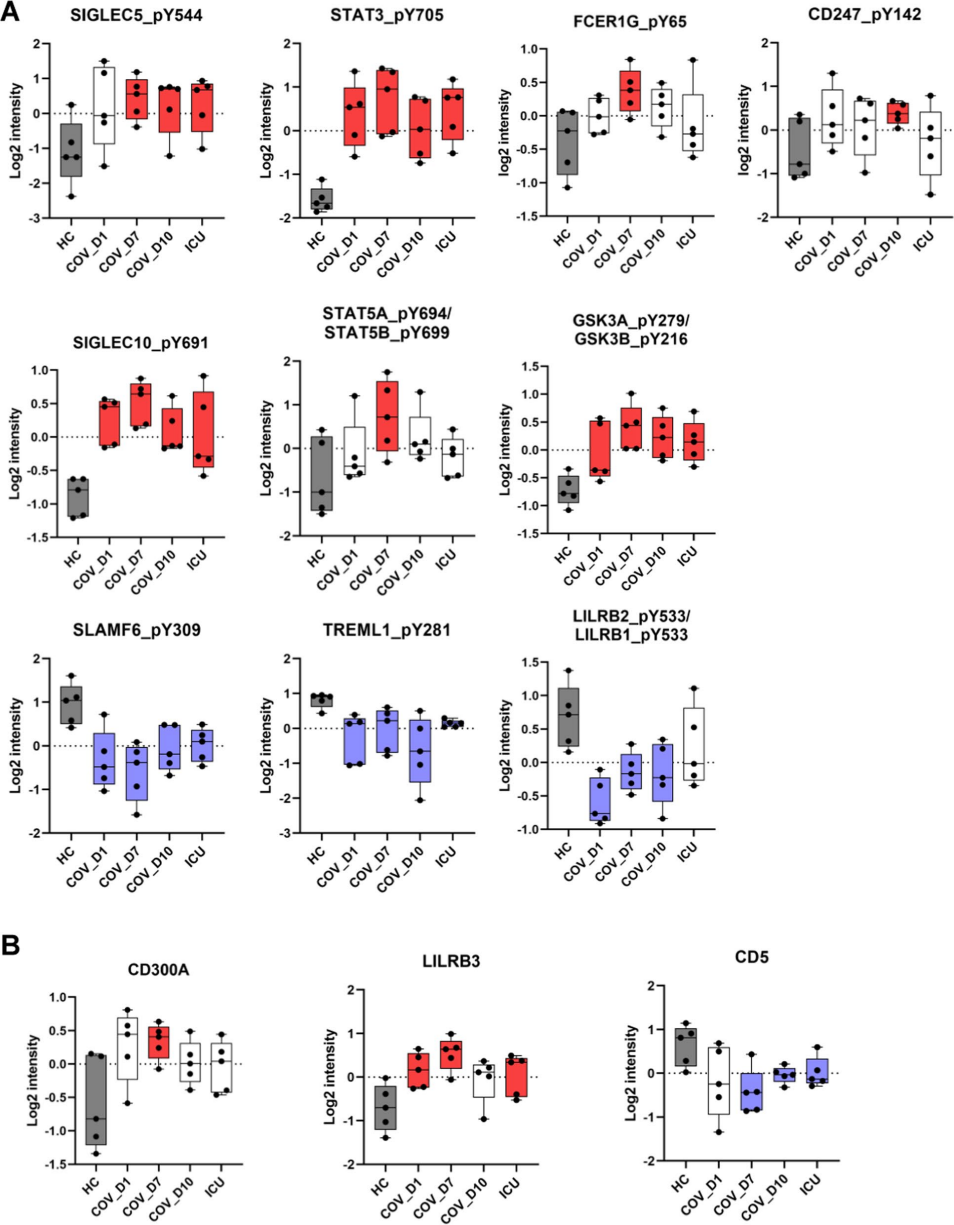
Significant changes in the Tyr phosphorylation (A) or expression (B) of key regulators of immune response. The boxplots are representative examples showing the dynamic changes in phosphorylation of the identified Tyr sites. Blue box: significantly lower than HC (p < 0.05 between the COV groupand HC, Student’s t-test), Red box: significantly higher than HC.

Because Tyr phosphorylation plays a central role in the proximal signaling by immunoreceptors and cytokines, we next focused our analysis on the significantly altered pTyr sites in COV compared to HC. More than 30 immune regulators were significantly over-phosphorylated on key Tyr residues, and an approximately equal number were under-phosphorylated (Fig. 5A). Of note, the activating Tyr residues in STAT3 and STAT5A/B were significantly over-phosphorylated (Figs. 5A and 6A), suggesting elevated cytokine signaling (vide infra).

Immune signaling is critically dependent on immunoreceptor Tyr-based regulatory motifs (ITRMs) [44]. The phosphorylation profile of the ITRMs, therefore, may be used to gauge the immune signaling landscape. Our MS analysis yielded phosphorylation data for numerous ITRMs (Fig. 5A, B). Of note, the immunoreceptor Tyr-based activation motifs (ITAMs) on CD3δ and CD3γ showed increased phosphorylation whereas the CD3ζ ITAMs showed a mixed phosphorylation pattern in the COV group, suggesting that TCR signaling is partially activated in the COVID-19 patients (Fig. 5B). In support of this assertion, LAT, an adaptor protein in TCR signaling, and NFAM1, an activator of the calcineurin/NFAT-signaling pathway, were phosphorylated more robustly in the COV PBMCs. Collectively, these data indicate that the COV patients possessed partially active circulating T cells despite having lymphopenia (Additional file 2: Table S1).

SIGLECs are a family of receptors that play an important role in immune self-tolerance and host defense [45]. SIGLECS expressed by different myeloid and lymphatic cells play a negative role in regulating the effector function of these cells. We found that the ITIM/ITSM phosphorylation in SIGLEC5 and SIGLEC10 was significantly increased in the COV cohort (Fig. 6A). Because the SIGLECs may be expressed in neutrophils, monocytes, NK cells or B cells, this suggests that immune tolerance in the COVID-19 patients may be mediated by enhanced sialic acid signaling in these cells. Furthermore, increased inhibitory signaling via the SIGLECs suggests compromised myeloid cell function.

NK cells, which may kill virus-infected cells, appeared to be significantly compromised in COV. The activity of NK cells is regulated by the SLAM family receptors containing ITSM sequences [46]. We found that the Tyr phosphorylation of several SLAM proteins, including SLAM6 and CD84, was significantly reduced. Moreover, phosphorylation of the ITAM in TYROBP/DAP-12, an adaptor for the activating NK cell receptor CD94/NKG2C, was down-regulated in both the COV and ICU cohorts (Fig. 5B). Furthermore, CD300A/MAIR-1, an inhibitory receptor in NK cells, was increased in expression (Figs. 5C and 6B). In contrast, the expression of PVR, which mediates NK cell adhesion and triggers NK cell effector functions, was significantly reduced (Fig. 5C). Collectively, these data suggest that NK cell receptor-mediated signaling and effector function were compromised by SARS-CoV-2.

### A critical role for the cytokine-JAK-STAT signaling pathway in COVID-19

**Fig. 7:**
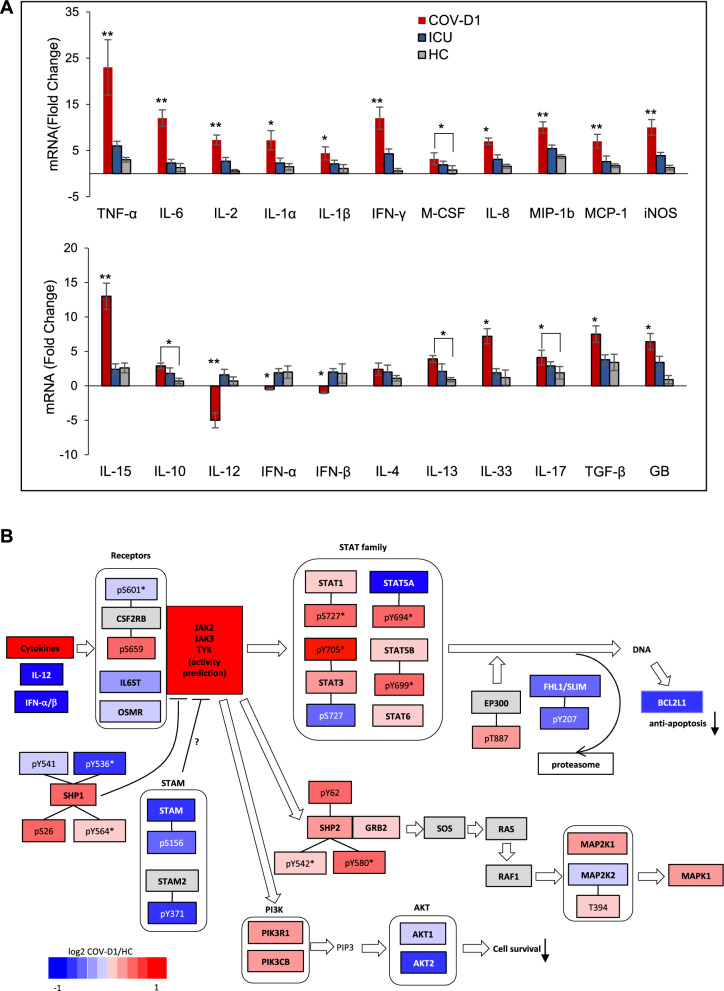
Cytokine-JAK-STAT signaling underlying COVID-19. **A** Differential expression of cytokines and chemokines in the COV-D1, ICU and HC PMBCs. Data shown were normalized to β-actin to calculate relative fold change in mRNA transcripts. Asterisks indicate significant difference between COV_D1 and ICU or HC unless otherwise specified. *p < 0.05, **p < 0.002, based on One-Way ANOVA (n = 5, 4 technical repeats). **B** A cytokine-JAK-STAT signaling network showing significant changes in protein expression or phosphorylation based on the MS data. The proteins and phosphosites are colored according to the COV-D1/HC ratio (red: upregulation, blue: downregulation). The nodes without sufficient data to calculate the ratio are colored grey. The phosphosites with an asterisk indicate activity-inducing residues

The cytokine release syndrome (CRS) or cytokine storm is a major cause of acute lung damage associated with patient mortality [47–52]. Because the cytokine storm is common in sepsis caused by SARS-CoV-2 and other pathogens [51, 53], we determined the mRNA levels of key proinflammatory cytokines and chemokines in the PBMCs of the COV and ICU cohorts in comparison to the healthy subjects. Except for IL-12, IFN-α, IFN-β, and IL-4, all examined cytokines/chemokines were significantly overexpressed in the COV cohort (Fig. 7A; Additional file 1: Fig. S5). In contrast, the ICU cohort showed only a few significantly upregulated, and by a much smaller degree. Therefore, SARS-CoV-2 infection elicited a much stronger cytokine storm in the COVID-19^+^ compared to the COVID-19^−^ sepsis patients (Additional file 2: Tables S1 and S2). In agreement with published data [6, 48, 54, 55], the cytokines most highly expressed included TNF-α, IL-6, IFN-γ, IL-2, and IL-15. In comparison, we observed a relatively low level of IL-1β [56]. Additionally, aside from cytokines, significant upregulation was noted for the chemokines MCP-1/CCL2 (recruiting monocytes and/or macrophages), IL-8/CXCL8 (a classic neutrophil chemoattractant), the macrophage inflammatory protein MIP-1b, and the macrophage colony-stimulating factor M-CSF.

These data agree with an important role for neutrophils, monocytes, and macrophages in the pathogenesis of COVID-19 [57, 58]. In support of this assertion, iNOS, which is frequently expressed by neutrophils, macrophages, and dendritic cells to produce NO, was significantly elevated. Inflammatory cytokines, including TNF-α and IFN-γ, may also induce NO production through the JAK-STAT1 signaling axis [48]. Moreover, we observed a continuous increase in transcript levels for cytokines/chemokines, with the exception of IL-12 and IL-18, from day 1 to day 7 and day 10 of ICU admission in the COV patients (Additional file 1: Fig. S5). Apart from one patient, all COVID-19 ICU patient ultimately succumbed to the disease, attributable at least in part to the unresolved cytokine storm.

It is intriguing that IL-12, which was slightly upregulated in the COV^−^ ICU patients, was significantly down-regulated in the COV^+^ cohort. IL-12, produced mainly by dendritic cells, macrophages, and lymphoblastoid cells, is required for the differentiation of Th1 cells and the activation of NK cells, both of which have been found to be defective in COVID-19 [55]. The defective NK cell signaling observed in our study (Fig. 5B) is consistent with reduced IL-12 expression as IL-12 plays a critical role in NK cell activation. In this regard, IL-18, which functions together with IL-12 in facilitating type 1 response, was also significantly down-regulated in the COV PMBCs (Additional file 1: Fig. S5). In contrast, the Th2 cytokines IL-4 and IL-10 were moderately increased, suggesting the CD4 ^+^ T cell lineage is skewed towards Th2 in COVID-19[55]. Besides IL-12, we found the transcripts of the type I interferons, IFN-α and INF-β, significantly reduced, suggesting impaired innate antiviral immunity.

Cytokines signal through the JAK-STAT pathway. As depicted in Fig. 4D, JAK2 and JAK3 were among the most highly activated kinases in COV PBMCs. Network analysis indicated that the JAK2/3 activation was reinforced by decreased phosphorylation/activation of SHP1 and STAM, which serve as negative regulators of cytokine signaling. Activation of the Janus kinases led to significant activation of STAT family of transcription factors, including STAT1, STAT3, STAT5, and STAT6 (Fig. 7B). STAT1 activation may underlie the significant changes in expression of proteins involved in regulating inflammation, complement, metabolism, and T cell signaling while the activation of STAT3, STAT5, and STAT6 may contribute to immune suppression. Collectively, the JAK-STAT pathway may signal to reduce the transcriptional activity of EP300 (i.e., increased T887 phosphorylation) and the expression of FHL1/SLIM, which is implicated in protein turnover and cardiomyopathy [59, 60]. JAK2/3 may also regulate cell proliferation through the SHP2-GRB2-SOS-RAS-RAF-MEK1/2 signaling pathway and survival through the PI3K-AKT2 pathway. The reduced expression of AKT1/2 suggests decreased cell survival, which, coupled with reduced BCL2L1 expression, may contribute to increased apoptosis and lymphopenia in COVID-19 [48, 55].

## Discussion

Compared to previous studies focusing on the plasma and PMBC transcriptome or proteome [28, 61, 62], our in-depth and quantitative MS analyses of both the proteome and phosphoproteome have provided unique insights into the molecular and systems basis of sepsis caused by SARS-CoV-2 or other pathogens. In addition to revealing significant differences in complement, coagulation, antigen presentation, and cytokine/chemokine expression, COVID-19 PMBCs are characterized by a reprogrammed kinome, leading to extensive rewiring of the phosphoproteome and the immune signaling network. Our work indicates that severe COVID-19 is marked by a partially active adaptive immune response, a compromised innate immune response, and an inbalance between antiviral and proinflammatory responses [55]. Key findings from our study are discussed briefly below.

### T cell and B cell activation despite lymphopenia

Despite a general reduction in lymphocyte count, robust T cell and B cell subsets have been reported in some patients [8, 63, 64]. A recent study also identified a profound activation of cytotoxic T cells in the blood of severe COVID-19 patients [23]. We observed that the COVID-19 ICU patients had activated T cell and B cell signaling characterized with activation of the tyrosine kinases LCK, LYN, and SYK. The T cell activation was also manifested by increased phosphorylation of some CD3 ITAMs and the TCR signaling proteins LAT and NFAM. However, decreased phosphorylation of approximately 2/3 ITAMs in CD3ζ suggests that the TCR activation is incomplete. It is likely that the TCR was activated by pMHC-I rather than pMHC-II, as MHC-II mediated antigen presentation was deficient in COVID-19 (Fig. 1F) [65]. Given that DCs are a major source of IL-12 production, the reduced IL-12 expression observed in the COV cohort may reflect a decrease in dendritic cells in critically ill COVID-19 patients. Likewise, the activated TCR and BCR signaling align with previous reports indicating that the magnitude and functional breadth of virus specific CD4 T cell and antibody responses are consistently higher in hospitalized patients [66].

### Enhanced inhibitory signaling and impaired innate immunity characterize COVID-19

Our work suggests that the impairment in innate antiviral immunity may involve at least three mechanisms. First, SARS-CoV-2 may reduce phagocytosis by promoting inhibitory signaling in phagocytes. Activation of the ITIM/ITSM-containing inhibitory receptor PECAM1 may contribute to reduced phagocytosis. Additionally, the activation of SIGLECs may block phagocytosis by a wide range of professional phagocytic cells, including neutrophils, monocytes, dendritic cells, and macrophages. By countering FcR-mediated phagocytosis, the sialic acid-SIGLEC axis may be exploited by SARS-CoV-2 to promote immune tolerance [67] or even contribute to antibody-dependent enhancement [68]. Putative sialic acid or galactose binding domains have been described in the Spike protein [69]. The broad-scale impairment of phagocytosis mediated by the ITIM/ITSM-containing inhibitory receptors explains why patients who developed neutralizing antibodies earlier in infection had a higher disease rate and worse outcomes than those who did not [70, 71]. Second, SARS-CoV-2 interferes with NK cell signaling and effector function. Several studies have reported the association of reduced NK cell number or cytotoxicity with disease severity [72]. Our study showed that both the number and signaling of NK cells through the SLAM family receptors were significantly reduced in the COV cohort. In addition to decreased FYN expression (Fig. 4A), which is responsible for SLAM receptor phosphorylation, IL-12 deficiency may contribute to reduced NK cell activity. Third, SARS-CoV-2 inhibits the production of the type I interferons IFN-α/β, in agreement with previous reports [73]. Because plasmacytoid dendritic cells (pDCs) are the major source of IFN-α/β, IL-12, and IL-18 production, reduced expression of these cytokines aligns with previous studies showing reduced pDC function and defective MHC-II-dependent antigen presentation in COVID-19 patients [65, 74]. Defects in antigen presentation, which is also critical for B cell function, may underlie the marked decrease in immunoglobins in the COV cohort (Fig. 3A).

### Targets for potential immunomodulatory therapy

In addition to providing valuable information on the adaptive and innate immune responses to SARS-CoV-2, we identified numerous potential therapeutic targets, especially protein kinases for COVID-19. Some of the kinase targets, including CK2, JAK2/3, SYK, and TYK2, have been identified in previous studies and reaffirmed in the current one. Importantly, the corresponding kinase inhibitors have shown promise in clinical trials [75–78]. Our study also identified numerous new kinases, such as DNA-PK, PKG1, ROCK1/, PKCδ, and GRK2, providing additional actional targets for the development of immunomodulatory therapies for COVID-19.

CK2 is likely a master regulator of immune response in COVID-19. CK2 may directly regulate viral RNA sensing and antiviral defense via the CK2-RIG1-TBK1-IRF3-IFN-α/β pathway. Our work suggests that CK2 may also regulate type I interferon response via the CK2-OPN-IFNα/β axis and inactivate NK cell effector function via CK2-OPN-IL-12. Furthermore, CK2 activation may affect the JAK-STAT pathway by phosphorylating JAK2 [79]. Therefore, inhibiting CK2 offers the potential to rejuvenate antiviral immunity while simultaneously mitigating the damaging effect of the cytokine storm. Notably, the CK2 inhibitor silmitasertib demonstrated suppression of SARS-CoV-2 infection in a cell model [41], and an anti-CK2 peptide improved clinical responses in COVID-19 patients with pneumonia [75].

SYK is another attractive target emerging from our MS analysis [80]. SYK activation in COVID-19 may be a double-edged sword [81]. On one hand, SYK is required for the B-cell receptor and FcR-mediated signaling pathways; on the other hand, it is involved in promoting inhibitory signaling in innate immune cells [82], compromising FcR-mediated phagocytosis of the virus or virus-infected cells. Nevertheless, a recent study has shown that SYK plays a critical role in memory effector T cell activation through macrophages in SARS-CoV-2 mRNA vaccination [83]. The therapeutic potential of targeting SYK was demonstrated in a recent study showing that the SYK inhibitor fostamatinib mitigated myeloid proinflammatory responses believed to contribute to the immunopathogenesis of severe COVID-19 [78].

Lastly, we propose that IL-12 and IL-18 supplementary therapy, either alone or in combination with proinflammatory cytokine blockade, may represent an effective strategy to combat severe COVID diseases. However, considering the wide range of cytokines that are significantly overexpressed, targeting a single cytokine-receptor pair may not be sufficient. For instance, there are at least 10 cytokines of the IL-6 family that can activate STAT3 [50]. This might explain why tocilizumab, an inhibitor of the IL-6 receptor, did not demonstrate significant benefits in terms of disease progression or survival for hospitalized patients with COVID-19 pneumonia in clinical trials [84, 85]. Indeed, anti-cytokine therapies for severe COVID-19 should be informed by detailed inflammatory profiling [86] and applied according to the underlying molecular mechanisms. Our work suggests that IL-12 and IL-18 should be considered in future cytokine-based therapies. Furthermore, JAK3, JAK2 and TYK2, which are highly activated in COVID-19, may be co-targeted with cytokine-modulatory therapy. Since these kinases transduce signals downstream of the cytokines, the corresponding inhibitor may help alleviate acute respiratory distress syndrome (ARDS) associated with cytokine release syndrome (CRS).

## Conclusions

The pathophysiology of SARS-CoV-2-induced ARDS closely resembles that of severe community-acquired pneumonia and sepsis caused by other viruses or bacteria [49], mirroring the comparison between the COV and ICU cohorts in the current study. Indeed, our in-depth proteome and phosphoproteome analysis of PBMCs from sepsis patients, whether positive or negative for SARS-CoV-2, revealed numerous common features, including compromised adaptive and innate immune responses to the pathogens.

A caveat of the current study is the small sample size (n = 5) which potentially makes it statistically underpowered (Additional file 1: Fig. S6). To address this concern, we mitigated this limitation by combining complementary evidence from multiple proteins and phosphosites to reinforce our analysis, avoiding reliance on a single protein or phosphosite. In addition to the array of potential therapeutic targets uncovered in our study, the extensive proteomics and phosphoproteomics datasets obtained herein may guide future investigations into the mechanisms underlying pneumonia and sepsis associated with viral or bacterial infections. Furthermore, they may facilitate the development of targeted immunomodulatory therapies for the treatment of these conditions [48]. Our comparative analysis of patient samples collected on different days of ICU admission highlighted the dynamic nature of the blood proteome and phosphoproteome in COVID-19. Specific changes or signature proteins/phosphoproteins identified can distinguish longitudinal disease states. Therefore, quantitative MS analysis of peripheral blood, being a readily available biospecimen, holds potential for monitoring disease progression and evaluating responses to COVID-19 therapies or vaccines over time [87].

### Supplementary Information

**Additional file 1: Figure S1.** An overview of sample processing for LC/MS-MS analysis. **Figure S2.** A flowchart describing how the proteins with dynamic changes in expression were extracted (related to Fig. 3A).  **Figure S3. **Prediction of STKs responsible for the increased phosphorylation of Phospho_Custer 2 on Day 1 (related to Fig. 3B). **Figure S4.** Clinical features of the COVID-19 samples collected on different days. **Figure S5.** Dynamic cytokine/chemokine expression in critically ill COVID-19+ patients during disease progression. **Figure S6.** Post-hoc power analysis for 10 pTyr sites used in Fig. 6A.

**Additional file 2: Table S1.** Subject demographic and clinical data. **Table S2.** Summary of clinical data. **Table S3**. Summary of cohort demographics. **Table S4.** A list of samples for TMT labeling and mass spectrometry analysis. **Table S5.** Mass spectrometer data collection parameters. **Table S6. **Proteome data used for analysis. **Table S7.** Phosphoproteome data used for analysis.

## Data Availability

The mass spectrometry proteomics data have been deposited to the ProteomeXchange Consortium via the PRIDE partner repository with the dataset identifier PXD024087.
