# Correction: Kinome and phosphoproteome reprogramming underlies the aberrant immune responses in critically ill COVID-19 patients

**DOI:** 10.1186/s12014-024-09484-7

**Published:** 2024-05-04

**Authors:** Tomonori Kaneko, Sally Ezra, Rober Abdo, Courtney Voss, Shanshan Zhong, Xuguang Liu, Owen Hovey, Marat Slessarev, Logan Robert Van Nynatten, Mingliang Ye, Douglas D. Fraser, Shawn Shun‑Cheng Li

**Affiliations:** 1 Department of Biochemistry, Western University, London, ON N6A 5C1 Canada; 2 Department of Pathology and Laboratory Medicine, Western University, London, Canada; 3 Departments of Medicine and Pediatrics, Western University, London, Canada; 4 CAS Key Laboratory of Separation Sciences for Analytical Chemistry, National Chromatographic R&A Center, Dalian Institute of Chemical Physics, Chinese Academy of Sciences (CAS), Dalian, 116023 China; 5 Lawson Health Research Institute, 750 Base Line Rd E, London, ON N6C 2R5 Canada

**Correction: Clinical Proteomics (2024) 21:13** 10.1186/s12014-024-09457-w

Following publication of the original article [1], the authors identified an error in the author name of Douglas D. Fraser.

The incorrect author name is: Douglas Fraser.

The correct author name is: Douglas D. Fraser.

The author group has been updated above and the original article [1] has been corrected.
