# Supplementary material for: Kinome and phosphoproteome reprogramming underlies the aberrant immune responses in critically ill COVID-19 patients
Source: Clin Proteomics. 2024 Feb 22;21:13. doi: 10.1186/s12014-024-09457-w (PMC10882830; doi:10.1186/s12014-024-09457-w)
Supplement: Supplementary file 1 — Additional file 1: Figure S1. An overview of sample processing for LC/MS-MS analysis. Figure S2. A flowchart describing how the proteins with dynamic changes in expression were extracted (related to Fig. 3A). Figure S3. Prediction of STKs responsible for the increased phosphorylation of Phospho_Custer 2 on Day 1 (related to Fig. 3B). Figure S4. Clinical features of the COVID-19 samples collected on different days. Figure S5. Dynamic cytokine/chemokine expression in critically ill COVID-19+ patients during disease progression. Figure S6. Post-hoc power analysis for 10 pTyr sites used in Fig. 6A. [file 12014_2024_9457_MOESM1_ESM.pdf]

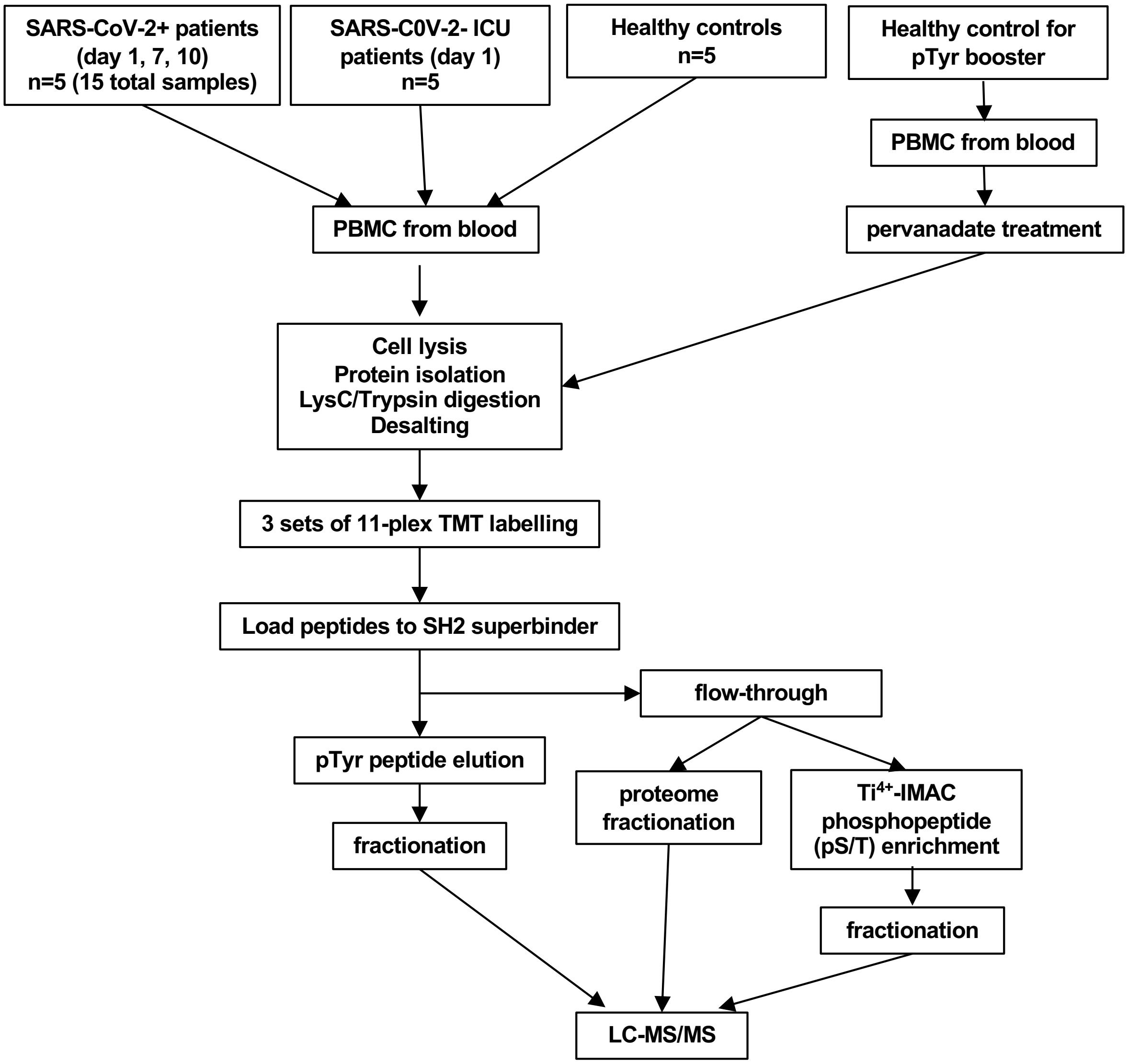

Figure S1. An overview of sample processing for LC/MS-MS analysis. PMBCs were isolated from patients or healthy control blood samples. Proteins were isolated and digested by LysC and trypsin. The peptides were labelled with 11-plex TMT labelling reagents in three batches/sets. The 11 peptide samples for each isobaric set were combined and incubated with SH2 superbinder (SH2S) agarose beads. The bound peptides were enriched for tyrosine-phosphorylated peptides. The flow-though materials were used for analysis of the proteome and the phosphoproteome (mainly for pSer and pThr peptides). The Ti^{4+}-IMAC resin was used for the phosphopeptide enrichment. Proteome and enriched phosphopeptides were fractionated using a high-pH fractionation kit prior to LC-MS/MS analysis.

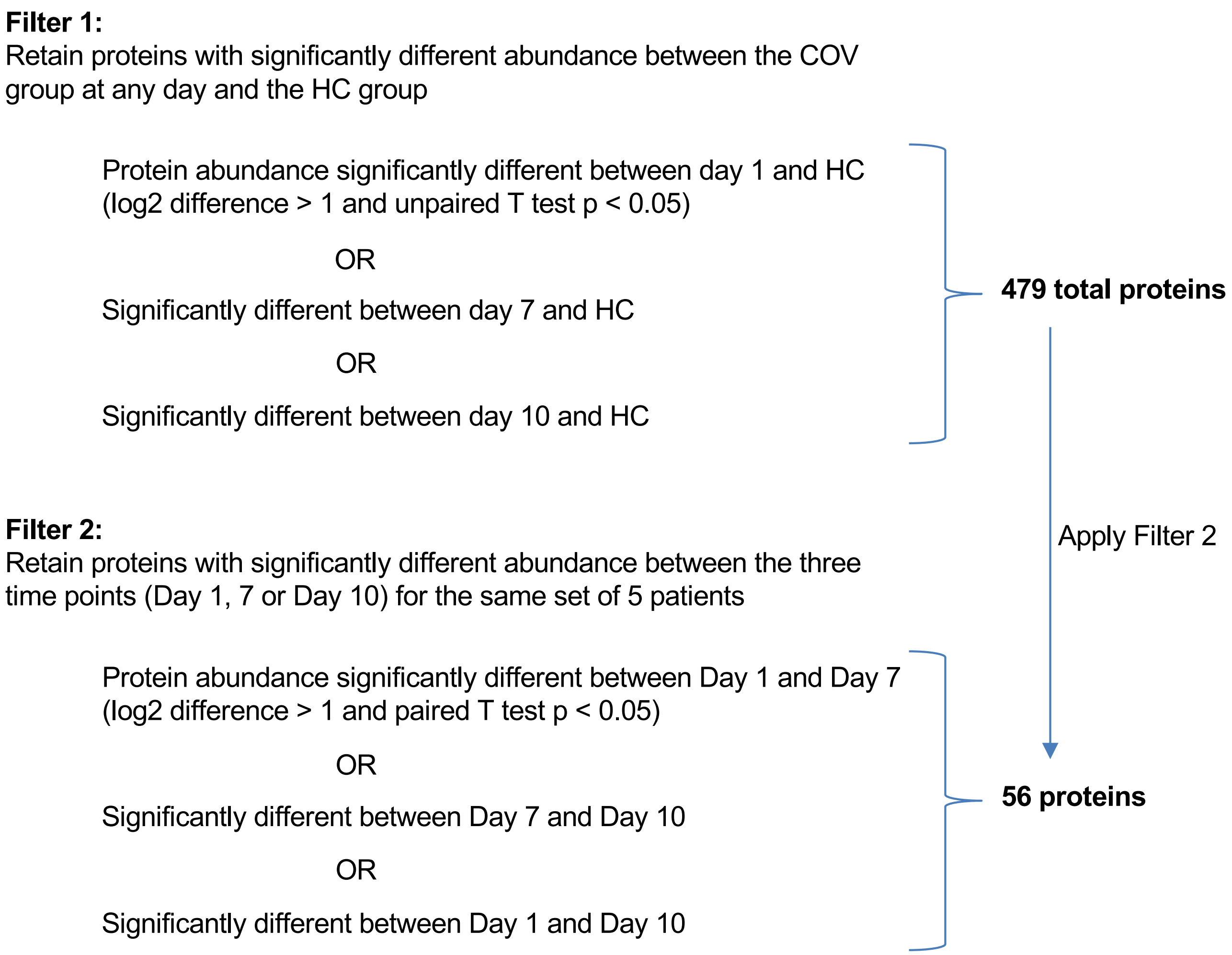

Figure S2: A flowchart describing how the proteins with dynamic changes in abundance were extracted (related to Fig. 3A)

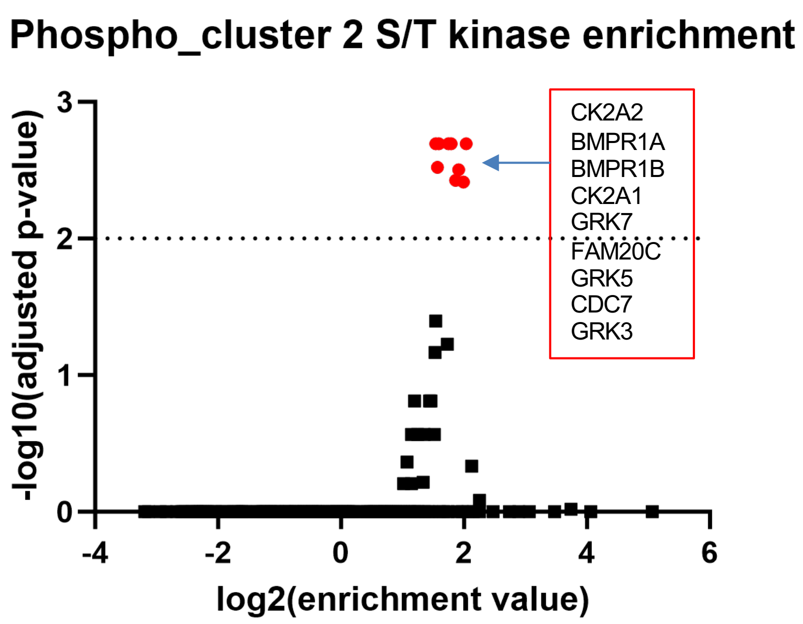

Figure S3. Prediction of STKs responsible for the increased phosphorylation of Phospho_Cluster 2 on Day 1 (related to Fig. 3B)

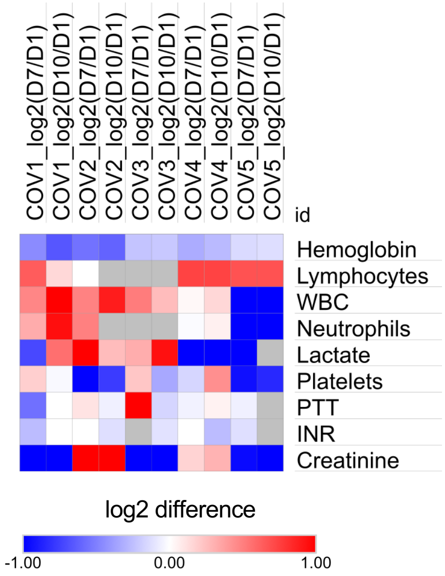

Figure S4. Clinical features of the COVID-19 samples collected on different days. Heat map of D7 and D10 data compared to D1 for the 5 COVID patient samples (additional details are provided in Table S1).

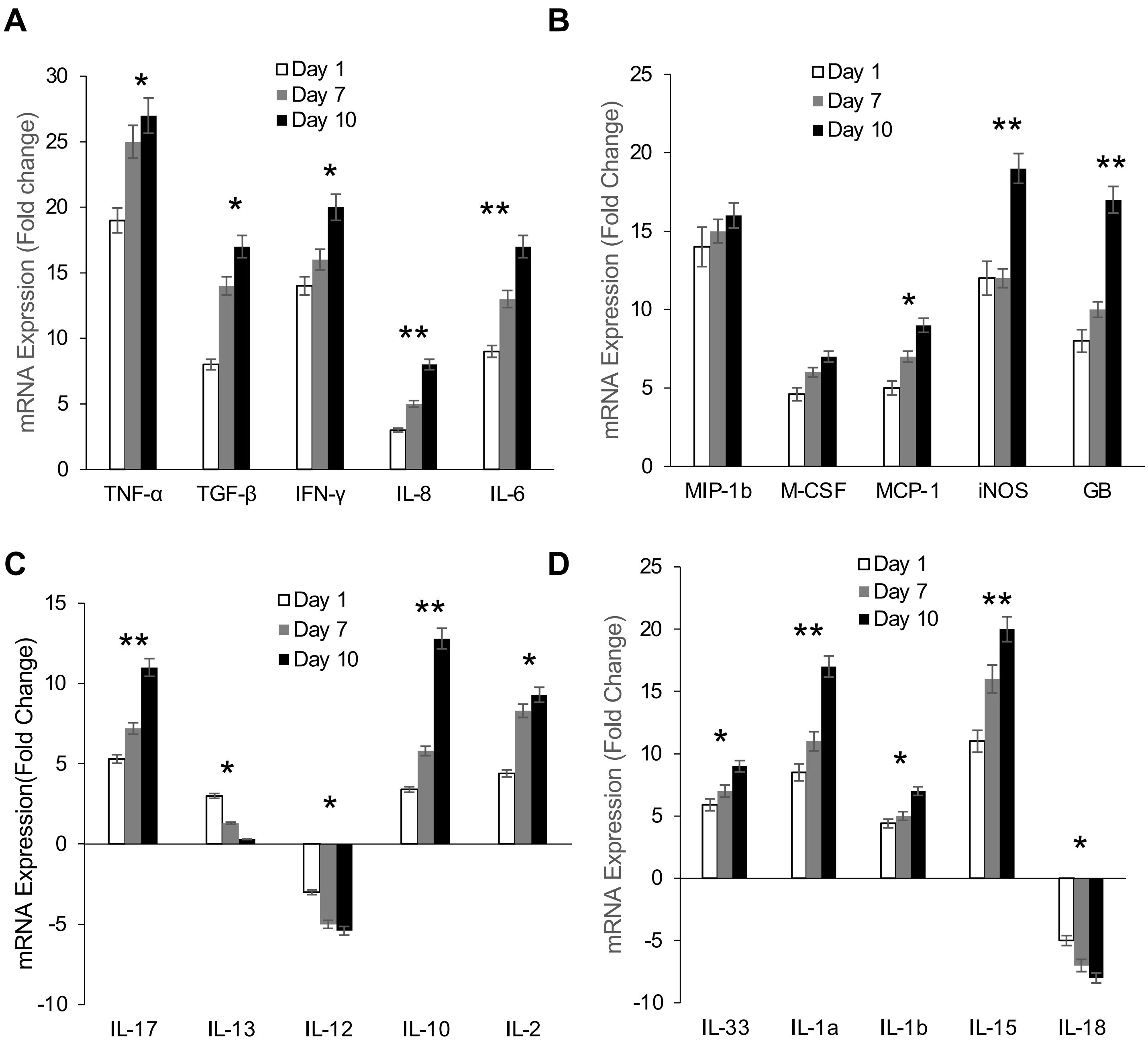

Figure S5. **Dynamic cytokine/chemokine expression in critically ill COVID-19+ patients during disease progression.** Cytokine and chemokine expression in PBMCs isolated on day 1, day 7 and day 10 after ICU admission. The qPCR data were normalized to b-actin to calculate relative fold change in mRNA expression. The significance of difference between the day 10 and day 1 samples was calculated based on One-Way ANOVA. *$p<0.05$, **$p<0.002$.

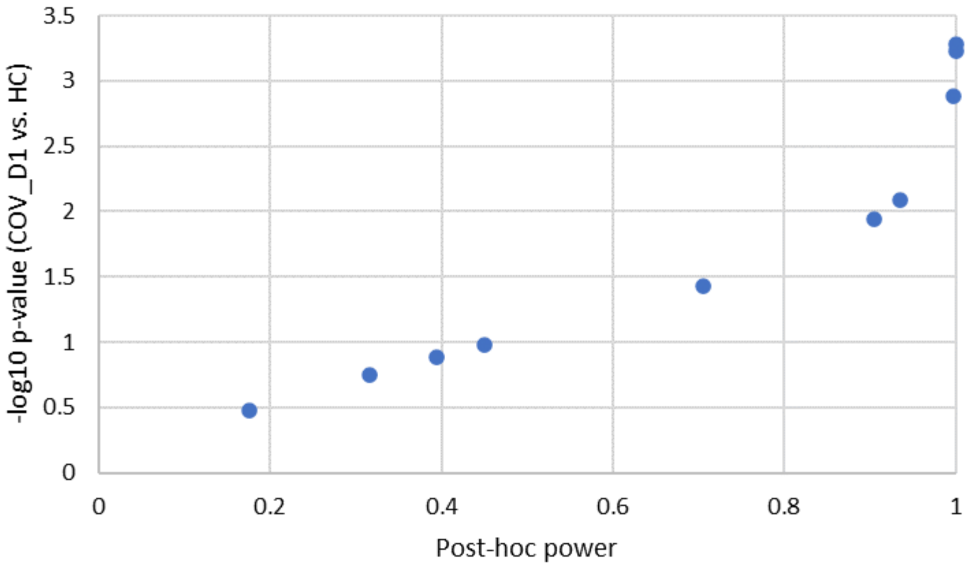

Fig. S6. Post-hoc power analysis for 10 pTyr sites used in Fig. 6A. The phosphosite intensities for COV_D1 and HC were used for calculation. The plot shows a correlation between the T-test p-values and the post-hoc statistical power.
